# Telecom-band–integrated multimode photonic quantum memory

**DOI:** 10.1126/sciadv.adf4587

**Published:** 2023-07-14

**Authors:** Xueying Zhang, Bin Zhang, Shihai Wei, Hao Li, Jinyu Liao, Cheng Li, Guangwei Deng, You Wang, Haizhi Song, Lixing You, Bo Jing, Feng Chen, Guangcan Guo, Qiang Zhou

**Affiliations:** ^1^ Institute of Fundamental and Frontier Sciences, University of Electronic Science and Technology of China, Chengdu 610054, China.; ^2^ School of Physics, State Key Laboratory of Crystal Materials, Shandong University, Jinan 250100, China.; ^3^ Shanghai Institute of Microsystem and Information Technology, Chinese Academy of Sciences, Shanghai 200050, China.; ^4^ CAS Key Laboratory of Quantum Information, University of Science and Technology of China, Hefei 230026, China.; ^5^ Southwest Institute of Technical Physics, Chengdu 610041, China.; ^6^ School of Optoelectronic Science and Engineering, University of Electronic Science and Technology of China, Chengdu 610054, China.

## Abstract

Telecom-band–integrated quantum memory is an elementary building block for developing quantum networks compatible with fiber communication infrastructures. Toward such a network with large capacity, an integrated multimode photonic quantum memory at telecom band has yet been demonstrated. Here, we report a fiber-integrated multimode quantum storage of single photon at telecom band on a laser-written chip. The storage device is a fiber-pigtailed Er^3+^:LiNbO_3_ waveguide and allows a storage of up to 330 temporal modes of heralded single photon with 4-GHz-wide bandwidth at 1532 nm and a 167-fold increasing of coincidence detection rate with respect to single mode. Our memory system with all-fiber addressing is performed using telecom-band fiber-integrated and on-chip components. The results represent an important step for the future quantum networks using integrated photonics devices.

## Teaser

On-chip multimode quantum memory is demonstrated at telecom band.

## INTRODUCTION

Photonic quantum memories (*1*, *2*), allowing reversible mapping of quantum states of light onto matter, are an indispensable component for quantum repeater based long-distance quantum communication (*3*) and distributed quantum networks (*4*–*7*). Optical waveguide–based photonic quantum memory devices connect with other integrated quantum devices, such as quantum light source (*8*–*10*), photonic circuit (*11*), and single-photon detector (*12*–*16*), which will open the way to integrated multifunctional quantum architectures. Rare-earth-ion–doped optical waveguides are excellent candidates for the development of on-chip quantum memory devices due to compactness, scalability, and enhanced light-matter interaction (*17*). Substantial efforts have been devoted to manufacturing on-chip storage devices through various methods, such as Ti^4+^ in-diffusion (*18*, *19*), focused ion beam milling (*20*), and femtosecond laser micromachining (FLM) (*21*). On the basis of devices from these methods, photonics quantum memories have already demonstrated in different systems (*22*–*39*). Recently, erbium ion–doped waveguides, thanks to the optical translation of ^4^I_15/2_ ↔ ^4^I_13/2_ at telecom band, have been used to realize integrated quantum memory at 1.5-μm wavelength (*24*–*27*, *40*–*42*). Toward a quantum repeater with large capacity, on-chip quantum memories with broadband (*22*–*24*, *29*) and multimode (*26*, *29*–*31*) properties have been demonstrated. For practical integrated quantum memory, an effective method is to develop fiber-integrated quantum storage chips (*25*, *37*), which can directly interconnect to current fiber system, thus facilitating the use of integrated devices in quantum networks. To date, such a fiber-integrated quantum storage chip with multimode capacity at telecom band has yet been demonstrated, especially a broadband storage of nonclassical light, which is a key step toward large-scale high-rate quantum networks compatible with existing telecom infrastructure.

Here, we demonstrate a telecom-band–integrated multimode storage in a Er^3+^:LiNbO_3_ waveguide. The laser-written waveguide fabricated by FLM is directly coupled to a single-mode fiber pigtail via an optical collimator at each end, guaranteeing the compatibility with fiber communication system. An on-chip quantum memory system is demonstrated with the fiber-pigtailed waveguide based on atomic frequency comb (AFC) protocol. With a 4-GHz-wide AFC, we experimentally realize a multimode quantum storage of 330 temporal modes of heralded single photon at 1532 nm with a 167-fold increasing of coincidence detection rate compared to the single mode. Our quantum memory chip paves the way for integrated memory–based quantum networks compatible with infrastructures of fiber communication.

## RESULTS

**Fig. 1.**
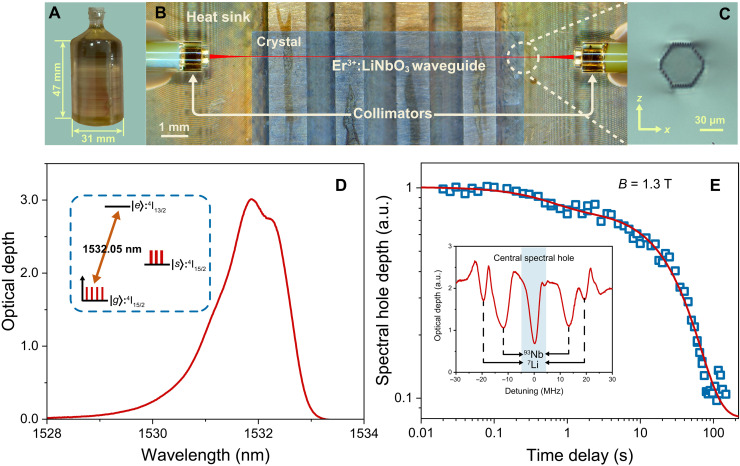
Preparation and calibration of Er^3+^:LiNbO_3_ waveguide. (**A**) Image of the bulk crystal of Er^3+^:LiNbO_3_. (**B**) Picture of fiber-integrated laser-written Er^3+^:LiNbO_3_ waveguide. The red line is for guiding eyes. Two optical collimators with single mode fiber-pigtails are used to couple with the waveguide. (**C**) Picture of the cross section of the waveguide. (**D**) Absorption spectrum of Er^3+^:LiNbO_3_ waveguide with level scheme (inset). (**E**) Spectral hole depth decays with the waiting time with a magnetic field of 1.3 T along the *c* axis. The experimental data are fitted by double exponential polynomial, from which we extract two lifetimes τ*_a_* and τ*_b_* of 0.55 ± 0.12 and 32.75 ± 2.31 s, respectively. The weights of τ*_a_* and τ*_b_* decays are 29 and 71%, respectively. The inset gives a 60-MHz-wide section of a spectral hole. a.u., arbitrary unit.

The storage device is a type III waveguide fabricated in a wafer of Er^3+^:LiNbO_3_ crystal by FLM (*43*). Figure 1A shows an image of the bulk crystal of Er^3+^:LiNbO_3_ used in our demonstration. The dopant concentration of Er^3+^ ions is 0.1 mol %. The bulk crystal is further divided into wafers with a dimension of 10 mm by 10 by 0.5 mm along the *x* × *y* × *z* axes (*z* cut), in which a laser-written waveguide is fabricated along the *y* axes. The coupling structure between the laser-written waveguide and single-mode fibers is shown in Fig. 1B. The Er^3+^:LiNbO_3_ crystal is glued on a copper heat sink, and two optical collimators with single-mode fiber pigtails are glued ~1.5 mm from the end face of the waveguide. Figure 1C shows the microscope image of the end face of our waveguide. The fiber-integrated device is placed in a dilution refrigerator with a temperature of 13 mK. A total optical transmission efficiency is 26% through the entire cryogenic setup. We measure its absorption spectrum, as shown in Fig. 1D. The measured inhomogeneous spectral width of Er^3+^ ions is 180 GHz at ^4^I_15/2_ ↔ ^4^I_13/2_ transition. The preparation of AFC requires frequency-selective population transfer of Er^3+^ ions from the ^4^I_15/2_ electronic ground level (∣*g*〉) through the ^4^I_13/2_ excited level (∣*e*〉) onto an auxiliary level (∣*s*〉), i.e., long-lived Zeeman sublevel (see the inset of Fig. 1D). We characterize the lifetimes of Zeeman sublevels of Er^3+^ ions by measuring the spectral hole burning (see note S2 for details of the setup). Figure 1E shows the decay of central spectral hole depth as a function of time delay, which is fitted by a double exponential polynomial curve Δα(*t*) = Δα*_a_e*^−(*t*/τ*_a_*)^ + Δα*_b_e*^−(*t*/τ*_b_*)^, where Δα*_a_* and Δα*_b_* are relative amplitudes of two population components present in the hole decay, τ*_a_* and τ*_b_* are 1/e population lifetimes of the short decay and the long decay, respectively. Our fitting results yield τ*_a_* = 0.55 ± 0.12 s with a weight of 29% and τ*_b_* = 32.75 ± 2.31 s with a weight of 71%, indicating the presence of two different classes of Er^3+^ ions in the lattice with different nuclear spin relaxation times (*44*). Moreover, because of the superhyperfine coupling of Er^3+^ electronic states to neighboring ^93^Nb and ^7^Li nuclear spins (*45*, *46*), two classes of side holes are observed in the hole burning spectrum (see the inset of Fig. 1E). In our experiment, we measure the side hole detunings under different magnetic fields. The fitted results of the detunings show a field-dependent detuning of 1.077 ± 0.039 and 1.487 ± 0.019 kHz/G for side holes arising from the ^93^Nb and the ^7^Li, respectively. More details on the measurement of side holes are given in note S2. With a magnetic field of 1.3 T, i.e., 13,000 G, the ^7^Li caused side holes with 20-MHz detuning coincide with the transparency regions of the AFC with 5-MHz teeth spacing, while the side holes with 12-MHz detuning caused by the ^93^Nb and the AFC is not well matched in our experiment.

**Fig. 2.**
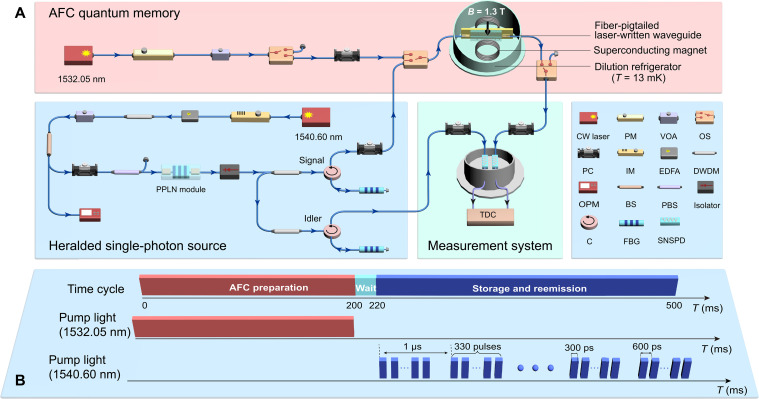
Experimental setup. (**A**) Schematics of the setup. Heralded single photon is obtained on the basis of cascaded SHG and SPDC processes in a fiber-pigtailed PPLN waveguide module. Signal photons are directly transmitted into a fiber-integrated laser-written Er^3+^:LiNbO_3_ waveguide placed in a dilution refrigerator. The signal photons are recalled with a preprogrammed storage time. Photons from signal and idler channels are detected by two SNSPDs. A time-to-digital converter (TDC) is used to analyze the detection signals by recording the single and coincidence counts of photons from both channels. CW laser, continuous wave laser; PM, phase modulator; VOA, variable optical attenuator; OS, optical switch; PC, polarization controller; IM, intensity modulator; EDFA, erbium-doped fiber amplifier; DWDM, dense wavelength division multiplexer; OPM, optical power meter; BS, beam splitter; PBS, polarization BS; C, circulator; FBG, fiber Bragg grating. (**B**) Experimental timing sequence. The sequence is continuously repeated during the experiment. A whole time period is 500 to 200 ms for preparing AFC, 20 ms for waiting, and 280 ms for storing signal photons.

Our experimental setup of multimode storage is sketched in Fig. 2A. It consists of the generation of heralded single photon, the preparation of AFC quantum memory, and the measurement system. Correlated photon pairs are generated by cascaded second-harmonic generation (SHG) and spontaneous parametric down conversion (SPDC) processes in a fiber-pigtailed periodically poled LiNbO_3_ (PPLN) waveguide module pumped by a series of light pulses at 1540.60 nm (*47*, *48*). For the single-mode storage, the PPLN module is pumped by a single laser pulse with a duration of 300 ps within a period of 1 μs. In addition, for the multimode storage, the PPLN module is pumped by 330 pulses with a separation of 600 ps within a period of 1 μs. A pair of photons at 1549.25 and 1532.05 nm are named idler and signal photons, respectively, and the bandwidth of photon pairs is 60 nm. The idler photons and signal photons are selected with bandwidths of 6.2 and 5.2 GHz by using two dense wavelength division multiplexers (DWDMs) followed by two fiber Bragg gratings (FBGs). The photons are detected by superconducting nanowire single-photon detectors (SNSPDs). A successful detection output of heralding idler photons heralds the presence of heralded signal photons. The twofold coincidences detection rates of our correlated photon pair source are 344.78 ± 0.59 and 49904.49 ± 7.06 Hz for the single-mode and the multimode storage, respectively. For the storage of the heralded single photon, the signal photons are sent to our fiber-integrated Er^3+^:LiNbO_3_ waveguide, in which a 4-GHz-wide AFC is prepared by using a continuous wave (CW) laser at 1532.05 nm modulated by a phase modulator (PM) with frequency chirped technology (*22*). The signal photons are mapped onto the AFC leading to a collective atomic excitation state, which can be described by a collective Dicke state (*49*).

$$|\psi \rangle =\frac{1}{\sqrt{N}}\sum_{j}^{N}c_{j}e^{i2{\pi \delta }_{j}t}e^{-{ikz}_{j}}|g_{1}\cdots e_{j}\cdots g_{N}\rangle$$ (1)

where *N* is the total number of ions; ∣*g_j_*〉 and ∣*e_j_*〉 represent the ground and excited states of ion *j*, respectively; *z_j_* is the position of ion *j*; *k* is the wavenumber of the light field; δ*_j_* is the detuning of the atom with respect to the laser frequency; and the amplitudes *c_j_* depend on the frequency and on the spatial position of atom *j*. The atomic collective state will rapidly dephase after absorption of photons. Owing to this periodic structure of the absorption profile, the atomic excitations will rephase and the signal photons will be recalled in the same mode after a predetermined time of 1/Δ (where Δ is the teeth spacing of the prepared AFC). The recalled signal photons are detected by a SNSPD through an optical switch (OS), which acts as a temporal gate to avoid the pump light for AFC preparation damage the SNSPDs and to ensure that recalled photons are not contaminated by noise photons stemming from the spontaneous decay of atoms with the excited state. Detection signals of the SNSPDs are analyzed by using a time-to-digital converter (TDC).

**Fig. 3.**
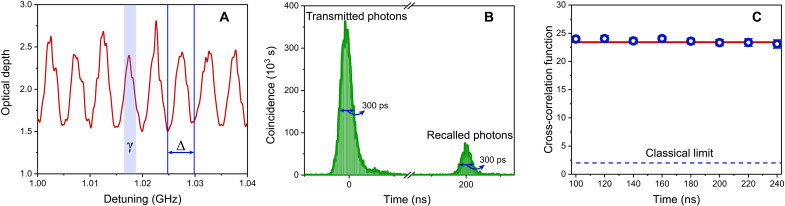
Characterization of the quantum memory. (**A**) A 40-MHz-wide section of the 4-GHz-wide AFC. The teeth spacing Δ is 5 MHz, corresponding to storage time of 200 ns. The linewidth of the teeth γ is ~2.5 MHz. (**B**) Temporal coincidence histogram between idler photons and signal photons after quantum storage—triggered by the system clock. The predetermined storage time is 200 ns, and the FWHM of a temporal mode is 300 ps. (**C**) Second-order cross-correlation function $g_{s,i}^{\left(2\right)}(\tau )$ between idler photons and signal photons with different predetermined storage times. The red solid line shows the value of 23.41 ± 0.04 of the $g_{s,i}^{\left(2\right)}(0)$ for the photon pair source before quantum storage. The $g_{s,i}^{\left(2\right)}(\tau )$ after the storage of signal photons still remains around 23. The detection windows of idler and signal channels are set to 600 ps. The classical threshold is given as a blue dashed line. The integration time is 1000 s, and the time bin width is 10 ps. The error bars are evaluated from the counts assuming Poissonian statistics.

We start to tailor a 4-GHz-wide inhomogeneous broadened profile of Er^3+^ ions into a periodical absorptive structure, i.e., AFC. Figure 3A shows a 40-MHz-wide section. The teeth spacing is 5 MHz, corresponding to a storage time of 200 ns. Consequently, we demonstrate the storage of nonclassical light with a large time-bandwidth product up to 800 (4 GHz × 200 ns). The signal photons with the full width at half maximum (FWHM) of 300 ps are sent to the AFC memory. Figure 3B shows the transmitted photons and the recalled photons with the predetermined storage time of 200 ns. A system efficiency is calculated to 0.59 ± 0.01% by the ratio of recalled heralded signal photon counts to all the input ones. Considering the transmission efficiency of the memory device and the spectral filtering of the input photons caused by the slightly smaller bandwidth of the AFC memory, an internal storage efficiency is calculated to 2.83 ± 0.03%. More details about internal storage efficiency can found in notes S5 and S6. The measured twofold coincidences detection rates are 344.78 ± 0.59 and 1.95 ± 0.04 Hz before and after storage, respectively. To assess the nonclassical property of the AFC quantum memory, we measure the second-order cross-correlation function $g_{s,i}^{\left(2\right)}(t)$

$$g_{s,i}^{\left(2\right)}(t)=\frac{P_{si}(t)}{P_{s}(t)\cdot P_{i}}$$ (2)

where *t* is the storage time, *P_si_*(*t*) is the probability of the twofold coincidence detection between idler photons and signal photons triggered by the system clock, and *P_s_*(*t*) (*P_i_*) is the probability of detecting the signal (idler) photons. The $g_{s,i}^{\left(2\right)}(0)$ for correlated photon pair source is 23.41 ± 0.04 before quantum storage [the red solid line in Fig. 3C, far exceeding the classical limit of 2 (*50*, *51*) and the blue dashed line in Fig. 3C]. We measure the values of $g_{s,i}^{\left(2\right)}(t)$ between idler and recalled signal photons at different storage times (*t*) from 100 to 240 ns, as shown in Fig. 3C. It shows that all values of $g_{s,i}^{\left(2\right)}(t)$ are around 23 even for the maximal storage time, which demonstrates that the AFC quantum memory preserves the nonclassical properties of correlated photon pairs. The slight changes of $g_{s,i}^{\left(2\right)}(t)$ are owing to the high signal-to-noise ratio of our AFC quantum memory, which is realized by synchronizing the storage process with the system clock (*48*). Furthermore, with the increased source pump power and repetition rate, we measure the heralded second-order autocorrelation function $g_{i:s,s}^{\left(2\right)}(t)$. The measured values are 0.23 ± 0.01 and 0.22 ± 0.06 before and after storage, respectively. We further measure the unheralded second-order autocorrelation function $g_{s,s}^{\left(2\right)}(t)$ of the signal photons. The measured values are 1.64 ± 0.01 and 1.58 ± 0.35 before and after storage, respectively. These results further indicate that the AFC quantum memory keeps the single-photon purity and the spectral purity of photons.

**Fig. 4.**
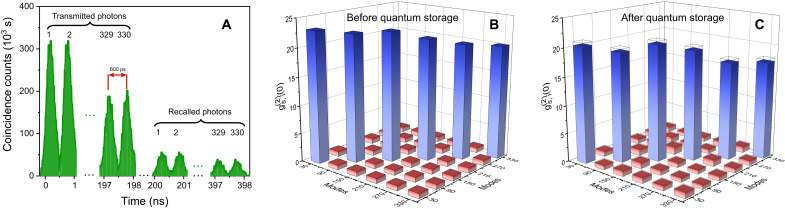
Results of the quantum storage of 330 temporal modes of heralded single photon. (**A**) Temporal coincidence histogram between idler photons and signal photons after quantum storage—triggered by the system clock. The FWHM width of each temporal mode is 300 ps, and a separation is 600 ps between adjacent modes. Storage time is set to 200 ns. (**B** and **C**) Second-order cross-correlation function between the idler and signal photons before and after quantum storage. The average second-order cross-correlation function for the 330 pairs of correlated temporal modes before quantum storage (after quantum storage) is 18.93 ± 0.04 (19.39 ± 0.01). The average second-order cross-correlation function of the uncorrelated temporal modes before quantum storage (after quantum storage) is 0.99 ± 0.01 (1.00 ± 0.01). The average value is acquired through Monte Carlo simulation. The detection windows of idler and signal channel of each temporal mode are set to 600 ps. The integration time is 1000 s, and the time bin width is 10 ps. The error bars are evaluated from the counts assuming Poissonian statistics.

We then establish a multiple temporal mode operation of on-chip quantum memory with the storage time of 200 ns. As shown in Fig. 4A, there are 330 distinguishable temporal modes of signal photons transmitted and recalled from the AFC quantum memory. The FWHM width of each temporal mode is 300 ps with a separation of 600 ps. The measured twofold coincidences detection rates are 49904.49 ± 7.06 and 326.70 ± 0.57 Hz before and after storage, respectively. Note that the dropping for the temporal modes with higher index number is due to the 50-ns dead time of SNSPDs. To assess the nonclassical properties of all stored modes throughout the AFC storage process, we obtain two sets of 330 × 330 values of $g_{s,i}^{\left(2\right)}(0)$ for photon pairs source and $g_{s,i}^{\left(2\right)}(t=200\;\mathrm{ns})$ between the idler and recalled signal photons, respectively. Figure 4 (B and C) presents parts of the second-order cross-correlation function corresponding to different temporal modes before and after quantum storage (the full 330 × 330 arrays of the second-order cross-correlation function are shown in note S7). We find that, for the 330 pairs of correlated temporal modes, the average $g_{s,i}^{\left(2\right)}(0)$ is 19.39 ± 0.01 and the average $g_{s,i}^{\left(2\right)}(t=200\;\mathrm{ns})$ is 18.93 ± 0.04, which demonstrates that the nonclassical properties of all temporal modes still maintain after the AFC quantum storage. The cross-talk between different temporal modes is assessed via measuring the second-order cross-correlation function between uncorrelated temporal modes (*52*). As expected, for all uncorrelated temporal modes, the average second-order cross-correlation function is 0.99 ± 0.01 and the one after quantum storage is 1.00 ± 0.01, which verifies that the cross-talk is negligible.

## DISCUSSION

In the present work, we have demonstrated an integrated multimode quantum memory based on a laser-written Er^3+^:LiNbO_3_ waveguide. A time-bandwidth product of 800 is achieved with a storage bandwidth of 4 GHz and a storage time of 200 ns. We have shown that the nonclassical correlations between idler photons and recalled signal photons at telecom band are well maintained after the retrieval. We have shown a quantum storage of 330 temporal modes of single photon at 1532 nm with a 167-fold increasing in coincidence detection rate compared to the single mode and measured the cross-talk between uncorrelated temporal modes, which is negligible. Our broadband multimode quantum storage will open the way to high-rate quantum network.

Despite these important results, several upgrades are needed toward a functional device for quantum network. The storage time of our demonstration is limited by the setups for the AFC preparation. For instance, the linewidth of our AFC pump laser is around 100 kHz with a frequency drift of MHz level, which prepares the AFC with a minimal teeth spacing of several MHz, resulting in a maximal storage time of hundreds of nanoseconds. Therefore, it needs to update the laser system by using an ultra-stable laser system to prepare the AFC (*53*, *54*). From the material side, it is necessary to extend the optical coherence time of the Er^3+^ ions in the waveguide (more details on optical coherence time, see note S3), which could be achieved by optimizing the dopant concentration of the Er^3+^ ions (*46*). On the other hand, the observed two classes of side holes influence the performance of the AFC memory, which are from the superhyperfine coupling of Er^3+^ electronic states to neighboring nuclear spins of the ^93^Nb and the ^7^Li. Ideally, the influence of these side holes could be eliminated by setting the transparency regions of the prepared AFC to coincide with the side holes with applying an optimized magnetic field (*55*). In our experiment, the storage time of 200 ns corresponds to an AFC with a teeth spacing of 5 MHz. With a magnetic field of 1.3 T, the transparency region of this AFC coincides with the side holes with the 20-MHz detuning from the neighboring ^7^Li. Hereby, the side holes caused by ^93^Nb may inescapably influence the performance of our memory. Possible method to avoid the side-hole effect is to implement quantum memory with other protocols (*56*) or to realize the AFC with specified storage times, such as 250 and 500 ns. Furthermore, thanks to the on-chip integration property of our demonstration, it is also feasible to further improve the performance of our Er^3+^:LiNbO_3_ memory, for instance, integrated with an on-chip microcavity for impedance-matched quantum memory (*57*, *58*) and with electrodes for the possible on-demand recall (*27*, *38*, *59*). It is also worth further exploring a possible Λ-like hyperfine energy-level system in our device to perform an on-demand recall based on the spin-wave storage (*60*, *61*), which could be a particular challenge in Er^3+^:LiNbO_3_ device because the Er^3+^ ions are the Kramers ions (*62*). Last, toward memories with larger capacities, a promising avenue is to combine multiple degrees of freedom. In addition to multiple temporal mode operation (*63*), more spectral channels could be prepared in the large inhomogeneous broadening of the Er^3+^:LiNbO_3_ waveguide with optical frequency combs (*48*). Moreover, more spatial channels could also be exploited in an on-chip quantum memory by fabricating several waveguides in the crystal.

In conclusion, our approach combines the reliability of a fiber-integrated device compatible with the fiber telecom infrastructure, the broadband multiplexed storage properties, and promising laser-written components. On the basis of our demonstration of multimode storage for heralded single photons at telecom wavelength with a 167-fold increasing compared to the single mode, next steps could be the storage of qubit, the creation of entanglement between remote memory chips, the implementation of feed-forward control, and the on-demand recall. The combination of photon pair sources and integrated memories with higher multimode capacity should allow the realization of a high-rate quantum repeater protocol toward a large-scale quantum network (*64*). Our result presents a fundamental building block towards the realization of a quantum repeater with large capacity, scalability, and compatibility of fiber communication infrastructure.

## MATERIALS AND METHODS

### Waveguide fabrication and fiber pigtailing

Our integrated storage device is a type III waveguide fabricated in a wafer of Er^3+^:LiNbO_3_ crystal (along crystal *y* axis) using FLM technology. The wafer of crystal—with a dimension of 10 mm by 10 mm by 0.5 mm along the *x* × *y* × *z* axes (*z* cut)—is cut from a bulk 0.1 mol % Er^3+^:LiNbO_3_ crystal, which is grown by Shanghai Institute of Optics and Fine Mechanics, Chinese Academy of Sciences. The waveguide is fabricated by a fiber femtosecond laser (FemtoYL-25, YSL Photonics). The central wavelength, pulse duration, and repetition rate are 1031 nm, 400 fs, and 25 kHz, respectively. A microscopic objective (Sigma Koki; 50×, numerical aperture = 0.67) is used to focus femtosecond laser into the wafer of Er^3+^:LiNbO_3_ crystal along the *z* axis (*c* axis). The focusing depth is around 180 μm, and the pulse energy of femtosecond laser on sample surface is around 0.26 μJ. The Er^3+^:LiNbO_3_ sample has been placed onto a motor-driven translation stage, and the scanning velocity along the crystal *y* axis is set as 3 mm/s. The polarization of femtosecond laser is perpendicular to the crystal *y* axis. As a result, a depressed-cladding waveguide is fabricated as shown in Fig. 1C. After FLM, thermal annealing (annealed at 200°C for 0.5 hours in air) has been applied to improve the guiding properties of waveguide. To realize on-chip quantum memory compatible with fiber communication infrastructure and to avoid challenges of free-space coupling, the waveguide is packaged into a fiber-pigtailed device. Two optical collimators (FCSS-G-155-1.8-A-1.1-025, Femto Technology, Xi'an) with single-mode fiber pigtails are coupling with the waveguide. The collimator is in cylinder shape with a length of 9 mm, a diameter of 1.4 mm, and a beam waist of around 50 μm. The operating wavelength range of optical collimators is 1510 to 1590 nm. Before gluing the collimators, they are held on a motorized stage with a moving resolution of 500 nm (A10-60, HUAVE). The distance between the collimators and the waveguide is monitored by using a microscope. The transmission efficiency of the coupling process is measured by using a laser light at 1570 nm—out of the absorption spectrum of the Er^3+^:LiNbO_3_ waveguide. The two optical collimators are glued on the heat sink when the measured transmission reaches maximum, i.e., a transmission efficiency of 40% in our demonstration. The loss is caused by the coupling mode mismatch, the waveguide propagation loss, imperfect waveguide facets, and so on. After mounting the fiber-pigtailed device into the dilution refrigerator and the cooling the temperature down to 13 mK, the total transmission efficiency through the entire cryogenic setup drops to 26% due to the fiber splicing and coupling misalignment in the cooling process. In perspective, we believe that one good strategy to improve the device transmission efficiency is to optimize the waveguide irradiation recipe to obtain a mode field distribution that better matches the collimator.

### AFC quantum memory

The fiber-pigtailed laser-written Er^3+^:LiNbO_3_ waveguide is cooled down to 13 mK in a dilution refrigerator (LD400, Bluefors) integrated with superconducting magnetic field of 1.3 T parallel to the *c* axis of crystals. We use a frequency-doubled laser diode (TOPTICA, CTL 1550) to generate the CW laser at 1532.05 nm for the AFC preparation. The light is modulated to a continuous frequency-chirped laser through frequency modulation, which is implemented using a lithium niobate PM driven by an arbitrary waveform generator (AWG) combining with the sideband-chirping technique. The frequency chirping extends from −2 to 2 GHz, thus efficiently preparing a 4-GHz-wide AFC memory. The pump power that we inject in the waveguide during the AFC creation is controlled to 28 μW by a variable optical attenuator (VOA), and the pump time is set to 200 ms by an OS. During this optical pump duration time, the frequency chirp cycle is repeated 1250 times. To ensure that the recalled photons are not contaminated by noise photons stemming from the spontaneous decay of atoms with the excited state, a wait time of 20 ms is applied after the optical pumping by controlling the state of the OS before the dilution refrigerator. Meanwhile, all $g_{s,i}^{\left(2\right)}(t)$ results are recorded by using a twofold coincidence detection triggered by the system clock. Following the wait time, it is 280 ms for storage and recall of the signal photons in the 500-ms measurement cycle. Before the signal photons enters the waveguide, the OS before the measurement system is turned off to protect the SNSPD.

### Heralded single-photon source

Correlated photon pairs are obtained by pumping a fiber-pigtailed PPLN waveguide module with a train of laser pulses through cascaded SHG and SPDC processes. The laser pulses are prepared by using a lithium niobite intensity modulator (IM) to externally modulate a CW laser at 1540.6 nm, which is generated from a narrow-linewidth semiconductor laser (PPCL300, PURE Photonics). The external modulation signals of the IM are derived from the pulsed electrical signals generated by an AWG and amplified to around *V*_π_ of the IM by a microwave amplifier. To ensure the optimal extinction ratio of light pulse, we use a 99:1 beam splitter (BS) combined with a photodetector to obtain a feedback electrical signal to the IM. These laser pulses are amplified by an erbium-doped fiber amplifier and then filtered out the fluorescence noise by a DWDM with a central wavelength at 1540.60 nm and a passband width of ~100 GHz. The power of laser pulses is attenuated and monitored by a VOA and a 90:10 BS with an optical power meter, respectively. To maximize the efficiency of phase matching before the PPLN waveguide module, a polarization BS and a polarization controller are used to manipulate the polarization of pump laser. The bandwidth of correlated photon pairs is ∼60 nm generated by the PPLN module. After the module, an isolator is used to reject the residual second-harmonic photons at 770 nm. Last, heralded signal photons at 1532.05 nm (heralding idler photons at 1549.25 nm) are spectrally filtered by a DWDM with a passband of ~100 GHz and an FBG with a passband of ~5.2 (~6.2) GHz. The idler photons are directly detected by a SNSPD (P-CS-6, PHOTEC Corp.; a detection efficiency of 59%, and a dark count of 70 Hz). Meanwhile, signal photons are sent to the fiber-integrated waveguide for storage and reemission and detected by another SNSPD (P-CS-6, PHOTEC Corp.; a detection efficiency of 76% and a dark count of 70 Hz). Detection signals are lastly analyzed by a TDC (quTAG, Qutools), which records the detection counts of the idler/signal channel and coincidence counts between the two channels triggered by the system clock (see note S4 for more details of heralded single-photon source).

### Calculation of $g_{s,i}^{\left(2\right)}(t)$, $g_{i:s,s}^{\left(2\right)}(t)$, and $g_{s,s}^{\left(2\right)}(t)$

The correlation between the idler and the signal photons is investigated by measuring the second-order cross-correlation function $g_{s,i}^{\left(2\right)}(t)$

$$g_{s,i}^{\left(2\right)}(t)=\frac{C_{si}(t)\times N}{C_{s}(t)\times C_{i}}$$ (3)

where *N* is the experimental trials for counting effective events with the integrated measurement time window, *C_si_*(*t*) is the twofold coincidence counts between idler and signal photons triggered by the system clock, and *C_s_*(*t*) (*C_i_*) is the detection counts of the signal (idler) photons.

The signal photons are split by a 50:50 fiber BS (FBS) and labeled as *s*_1_ and *s*_2_ for different outputs of FBS. The single-photon purity is evaluated by measuring the heralded second-order autocorrelation function $g_{i:s,s}^{\left(2\right)}(t)$

$$g_{i:s,s}^{\left(2\right)}(t)=\frac{C_{i,s_{1},s_{2}}(t)\times C_{i}}{C_{i,s_{1}}(t)\times C_{i,s_{2}}(t)}$$ (4)

where *C*_*i*,*s*_1_,*s*_2__(*t*) is the threefold coincidence counts among idler photons, *s*_1_ and *s*_2_, and *C*_*i*,*s*_1__(*t*) [*C*_*i*,*s*_2__(*t*)] is the twofold coincidence counts between idler photons and *s*_1_ (*s*_2_). All the coincidence measurements are triggered by the system clock.

The spectral purity is characterized by measuring the unheralded second-order autocorrelation function $g_{s,s}^{\left(2\right)}(t)$ of the signal photons

$$g_{s,s}^{\left(2\right)}(t)=\frac{C_{s_{1},s_{2}}(t)}{A_{s_{1},s_{2}}(t)}$$ (5)

where *C*_*s*_1_,*s*_2__(*t*) is the twofold coincidence counts between *s*_1_ and *s*_2_ and *A*_*s*_1_,__*s*_2__(*t*) is the twofold accidental coincidence counts of signal photons *s*_1_ and *s*_2_. All the coincidence measurements are triggered by the system clock.

For all the coincidence measurements here, the width of coincidence window is 600 ps. The integrated measurement time is 1000 s and the time bin width is 10 ps for the measurement of the $g_{s,i}^{\left(2\right)}(t)$. The integrated measurement time is 1000 s for the measurement of the $g_{i:s,s}^{\left(2\right)}(0)$ and $g_{s,s}^{\left(2\right)}(0)$, and the integrated measurement time is >20 hours for the measurement of $g_{i:s,s}^{\left(2\right)}(t=200\;\mathrm{ns})$ and $g_{s,s}^{\left(2\right)}(t=200\;\mathrm{ns})$. The time bin width is 100 ps for the measurement of $g_{i:s,s}^{\left(2\right)}(t)$ and $g_{s,s}^{\left(2\right)}(t)$.

## Supplementary Materials

### This PDF file includes:

Supplementary Text Notes S1 to S7 Figs. S1 to S6 Tables S1 and S2 References
