# Supplementary material for: Telecom-band–integrated multimode photonic quantum memory
Source: Sci Adv. 2023 Jul 14;9(28):eadf4587. doi: 10.1126/sciadv.adf4587 (PMC10348679; doi:10.1126/sciadv.adf4587)
Supplement: Supplementary file 1 — Supplementary Text Notes S1 to S7 Figs. S1 to S6 Tables S1 and S2 References [file sciadv.adf4587_sm.pdf]

Science Advances
AAAS

Supplementary Materials for

Telecom-band-integrated multimode photonic quantum memory

Xueying Zhang *et al.*

Corresponding author: Feng Chen, drfchen@sdu.edu.cn; Qiang Zhou, zhouqiang@uestc.edu.cn

Sci. Adv. **9**, eadf4587 (2023)
DOI: 10.1126/sciadv.adf4587

This PDF file includes:

Supplementary Text
Notes S1 to S7
Figs. S1 to S6
Tables S1 and S2
References

Supplementary Text

Note S1: Absorption spectrum of Er^{3+}: $LiNbO_3$

Figure S1 shows the experimental setup of measuring the absorption spectrum of Er^{3+}:$LiNbO_3$ waveguide. A broad spectrum light with the bandwidth of 100 nm (the wavelength ranges from 1470 nm to 1570 nm) - from an erbium-doped fiber amplifier (EDFA) - is used to pump the Er^{3+}:$LiNbO_3$ waveguide. The optical power and the polarization of the input light is controlled by a variable optical attenuator (VOA) and a polarization controller (PC). We set the pump power to 10 μW, and the spectrum is measured by a spectrum analyzer with a resolution of 0.02 nm. The optical depth corresponding to each wavelength is obtained by the formula: $P_{out} = P_{in}e^{-\alpha L}$ (*65*), where P_{out} and P_{in} are the optical power after and before the waveguide, α is the absorption coefficient, L is the length of the waveguide, and αL represents the optical depth. The absorption spectrum is shown in Fig. 1C of main text and the full width at half maximum (FHWM) of inhomogeneous spectral width is around 180 GHz.

Note S2: Zeeman-sublevel lifetime of Er^{3+}:$LiNbO_3$

We perform spectral hole burning (SHB) to investigate the lifetime of Zeeman sublevels of Er^{3+}:$LiNbO_3$ at a temperature of 13 mK. The experimental setup is shown in Fig. S2A. A continuous-wave (CW) laser with a wavelength of 1532.05 nm is split into two beams by a 50:50 beam splitter (BS). One is utilized as excitation light to prepare a persistent spectral hole, and another one is utilized as probe light to detect the hole. We shift the frequency of the excitation light by a 1.5 GHz via the phase modulator (PM1) with serrodyne modulation (*66*). The excitation time is set to 200 ms by the optical switch (OS1). The OS2 is used to control the time-delay between excitation light and the probe light entering the waveguide. After a varying time-delay, the spectral hole is detected by linearly varying the frequency of the laser light. Frequency sweeps are achieved by the PM2 with serrodyne modulation. The time of frequency sweeps is 10 μs controlled by an acousto-optic modulator (AOM), and the range is over a 100-MHz-wide frequency window centered at the excitation frequency. During the time for hole preparation, the OS3 are used to avoid the pump laser entering the photodetector (PD). Figure S2B shows the optical depth of Er^{3+} ions over a 60-MHz-wide spectral range centered at 1532.05 nm after applying a magnetic field of 1.3 T. The observed side holes around the central hole result from strong coupling of the Er^{3+} electronic states to neighboring ^{93}Nb and ^{7}Li nuclear spins (*24*, *46*). The central spectral hole broadened to a FWHM of 4 MHz. The side holes broadened to a FWHM of 6 MHz as the ^{93}Nb relaxed and then to 3 MHz as the ^{7}Li relaxed. The detuning of side holes arising from ^{93}Nb and ^{7}Li are ~12 MHz and ~20 MHz, respectively. To characterize the level splitting influenced by magnetic field, we plot the field-dependent detuning of side-holes at

different magnetic field with B//c (see Fig. S2C). Fits yield values of ^{93}Nb of 1.077 ± 0.039 kHz/G and ^{7}Li of 1.487 ± 0.019 kHz/G.

Note S3: Optical coherence time of Er^{3+}:$LiNbO_3$

The upper limit of the storage time for quantum memory could be the optical coherence time of the Er^{3+}:$LiNbO_3$, which can be obtained by measuring two-pulse photon echoes. The experimental setup for measuring the two-pulse photon echoes is shown in Fig. S3A. In the experiment, a CW laser with a wavelength of 1532.05 nm is modulated into two-pulse sequence by using an AOM, i.e., a π/2 pulse and a π pulse. The peak power of the pulses is about 8 mW. Figure S3B shows the photon echo area as a function of the time delay t_{12} between the π/2 pulse and the π pulse, which is fitted by $A(t_{12}) = A_0 \exp[-2(2t_{12} / T_2)^x]$, where A_0 is the echo area at $t_{12} = 0$; T_2 is the fitted optical coherence time; x describes the decay shape (*67*). With the measured experimental results, a fitted T_2 of 90.34±1.13 μs is obtained with a fitted x of 1.93.

Note S4: Properties of heralded single photon source

Figure S4A shows the single detection rates from signal and idler side, which are well fitted with a quadratic polynomial curve. Different single detection rate between the signal and idler channel is originated from the different measured efficiency and bandwidth of the filters. The measured efficiency of idler (signal) channel is 17% (13%), including the coupling efficiency of 77% (77%) for the fiber coupling into a fiber-pigtailed periodically poled $LiNbO_3$ (PPLN) waveguide module, the transmission efficiency of 39% (25%) from the output of PPLN to the input of SNSPD, and the detection efficiency of 62% (76%) for SNSPD. The bandwidth of the FBG with the central wavelength of idler photons (signal photons) is ∼ 6.2 GHz (5.2 GHz). Figure S4B shows the two-fold coincidence detection rate between the idler photons, signal photons triggered by the system clock corresponding to different pump power. The two-fold coincidence detection rate triggered by the system clock is available to eliminate the effects of accidental coincidence counts, which avoids imperfect extinction ratio of light pulse from increasing the coincidence detection rate. Figure S4C shows the second-order cross-correlation function ($g_{s,i}^{(2)}(0)$) as a function of the detected probability of idler photons (P_{idler}), which are well fitted with an inversely proportional curve. In the main text, we set the pump power to 36 μW. For single mode storage, the measured single detection rates of idler and signal channels are 3652.22±1.91 Hz and 2267.77±1.52 Hz, respectively. For multimode storage, the measured single detection rates of idler and signal channels are 466643.66±68.31 Hz and 241836.93±15.55 Hz, respectively. The two-fold coincidences detection rates are 344.78±0.59 Hz and 49904.49±7.06 Hz for the single mode storage and multimode storage, respectively. For single mode storage, the detected probability of idler (signal) photons, i.e., P_{idler} (P_{signal}), is measured to 0.63% (0.41%) with a $g_{s,i}^{(2)}(0)$ of

23.41±0.04. Combining a measured efficiency of 17% and the P_{idler}, an intrinsic generation probability of idler photons is calculated to 3.71% in the waveguide. If signal-to-noise-ratio (SNR) of correlated photon pairs is large enough, the theoretical value of $g_{s,i}^{(2)}(0)$ should be 27.98 (*48*), while the experimentally measured value of $g_{s,i}^{(2)}(0)$ is 23.41. The main factor is the existence of spontaneous Raman scattering noise photons generated from the PPLN waveguide module (*47*). The main parameters of the PPLN waveguide module are shown in Table S1.

Note S5: The internal storage efficiency of AFC

To analyze the internal efficiency of our quantum memory based on the fiber-pigtailed laser-written Er^{3+}:$LiNbO_3$ waveguide, we define a system efficiency as $\eta_s = N_{out} / N_{in}$, where N_{out} and N_{in} is the detection rate of the recalled signal photons and input signal photons on condition with heralding idler photons. The system efficiency contains the transmission efficiency (η_t), the efficiency of spectral filtering, and the internal storage efficiency (η_i). The transmission efficiency includes coupling efficiency between two ends of the waveguide and two optical collimators with single mode fiber-pigtails and a total fiber transmission efficiency in and out of the dilution refrigerator. To measure η_t , the light centered at 1570 nm - far away from the absorption profile of Er^{3+}:$LiNbO_3$ - is transmitted into the cryostat at the cooling temperature of 13 mK. Then we evaluated η_t =26% by the ratio of output power to input power. The efficiency is 77% due to spectral filtering of the input photons (5.2 GHz bandwidth) caused by the slightly smaller bandwidth of the AFC memory (4 GHz). Finally, the internal efficiency is calculated as $\eta_i = 1.3 \times \eta_s / \eta_t$. For example, when we set the storage time to 200 ns, we detect the coincidence counts triggered by system clocks before (after) storage is 3.4×10^5 Hz (2.0×10^3 Hz) among input (recalled) signal photons, idler photons and trigger signals for the integrated time of 1000 s. As a result, η_s is calculated as 0.59±0.01% and η_i is 2.83±0.06%. Table S2 shows that the internal storage efficiencies with different storage times range from 100 ns to 240 ns. In the experiment, the storage time is set by changing the properties of the laser for AFC preparation. An internal storage efficiency of 4.74±0.04% is obtained at a storage time of 160 ns with the magnetic field of 1.3 T, thanks to the ^{93}Nb caused 12 MHz detuning side holes coincide with the transparency regions of the AFC with a teeth spacing of 6 MHz.

Note S6: The optimization of storage efficiency

The storage efficiency for our memory device is related to the magnetic field and to the pump power for AFC preparation. The magnetic field strength directly influences relaxation dynamics, the lifetime of Zeeman sublevels, side holes detuning, linewidth broadening and so on (*24, 62, 68, 69*). The power of the AFC pump light greatly affects the non-zero background absorption of AFC by instantaneous spectral diffusion and light-induced two-level systems excitation (*45*). To obtain

an optimized storage efficiency with the storage time of 200 ns, we optimize the magnetic field and the pump power in our experiment. Figure S5A shows the internal storage efficiency of AFC with the different magnetic field strength. Figure S5B shows the internal storage efficiency of AFC with the different AFC pump power. With an optimized AFC pump power of 28 μW and an optimized magnetic field of 1.3 T, the highest internal storage efficiency of 2.83±0.06% is obtained with the storage time of 200 ns.

Note S7: 330 × 330 array of $g_{s,i}^{(2)}(t)$ among 330 temporal modes

For the multimode storage, there are 330 temporal modes generated with a peak separation of 600 ps within a period of 1 μs. We label each temporal mode of the signal channel as S_m (m=1, 2, ..., 330) and the idler channel as I_n (n=1, 2, ..., 330). By measuring coincidences between S_m and I_n, we calculate a 330 × 330 matrix of second-order cross-correlation function. The values of second-order cross-correlation function among 330 single photon modes before/after quantum storage are shown in Fig. S6 A/B. The non-classical nature of the AFC quantum memory and crosstalk between different temporal modes are assessed by the second-order cross-correlation function of correlated modes (m=n) and uncorrelated modes (m≠n), respectively.

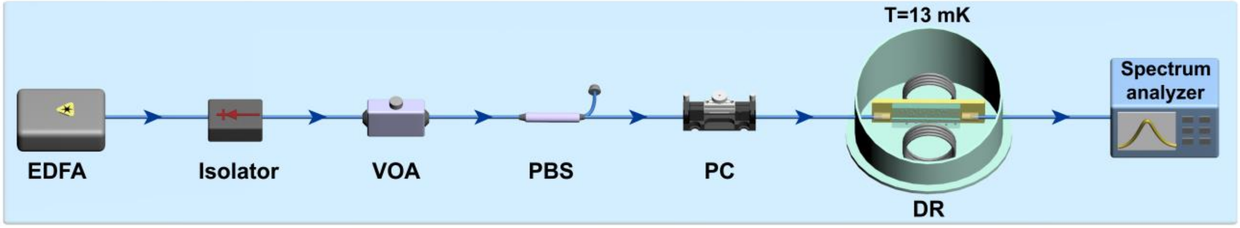

Fig. S1. Experimental setup for measuring the absorption spectrum of Er^{3+}:$LiNbO_3$. EDFA: erbium-doped fiber amplifier; VOA: variable optical attenuator; PBS: polarization beam splitter; PC: polarization controller; DR: dilution refrigerator. The Er^{3+}:$LiNbO_3$ waveguide is placed in a dilution refrigerator with a cooling temperature of 13 mK.

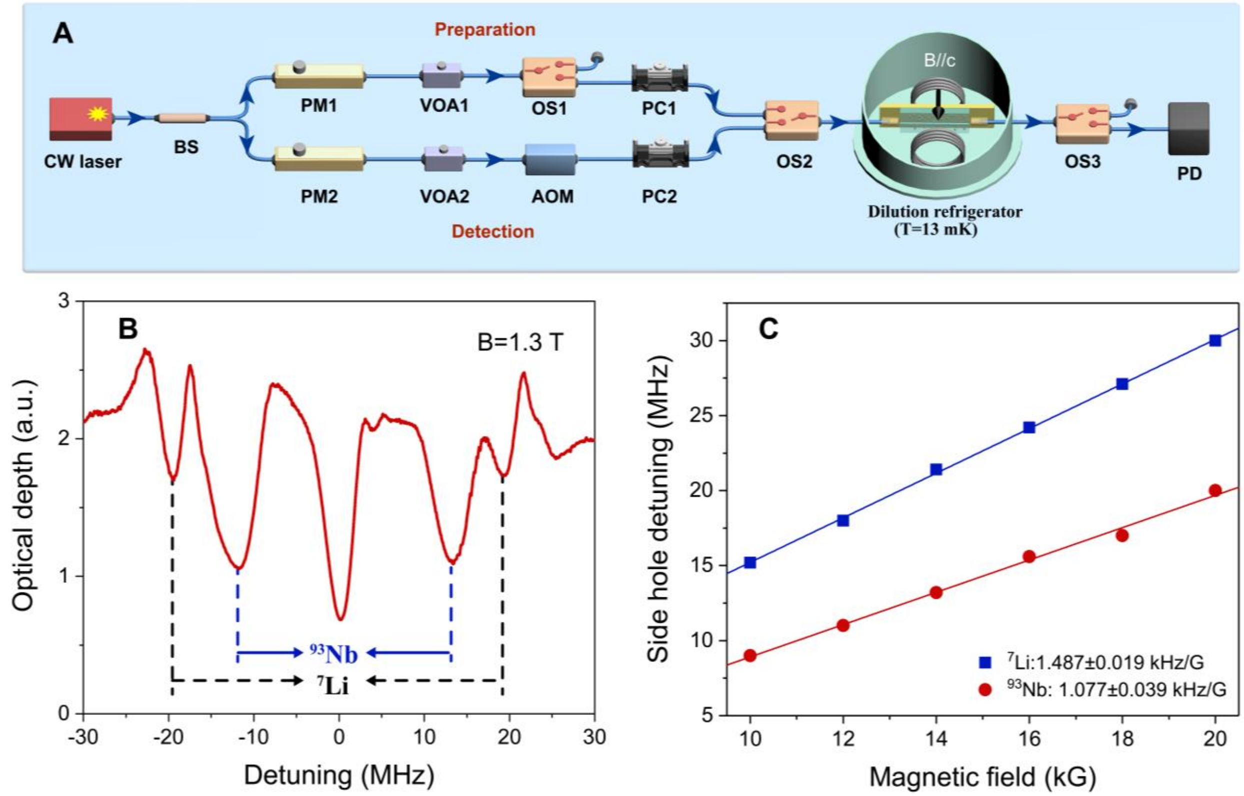

Fig. S2. Spectral hole burning. (**A**) The experimental setup. CW laser: continuous-wave laser; BS: beam splitter; PM: phase modulator; VOA: variable optical attenuator; OS: optical switch; AOM: acousto-optic modulator; PC: polarization controller; DR: dilution refrigerator; PD: photodetector; DOS: digital oscilloscope. (**B**) The optical depth of spectral hole centered at 1532.05 nm in the Er^{3+}:$LiNbO_3$ waveguide at 13 mK with a magnetic field of 1.3 T along the c-axis. (**C**) Side hole detunings of ^{7}Li and ^{93}Nb as a function of magnetic field.

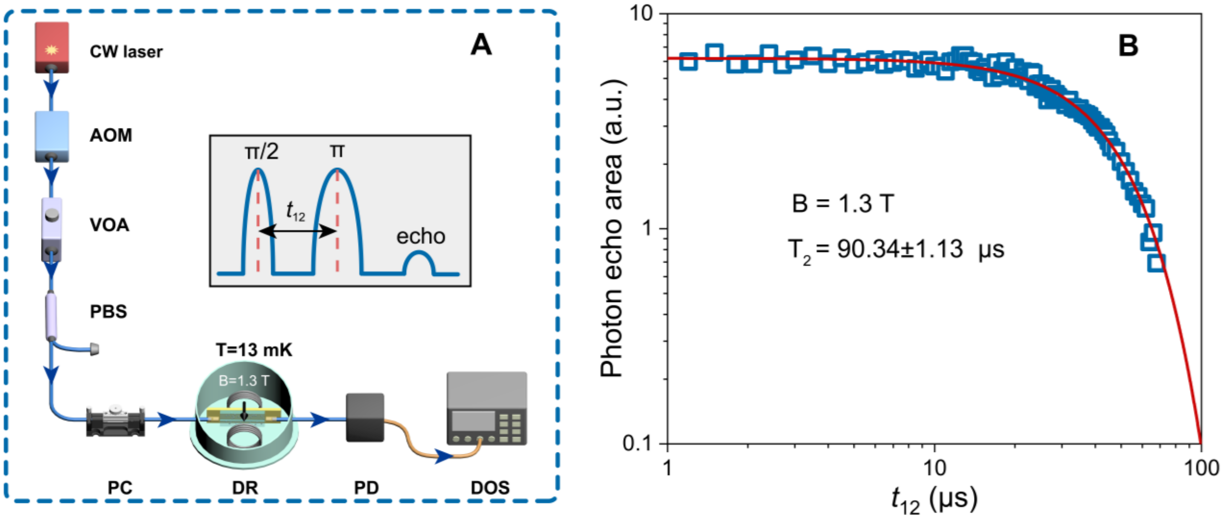

Fig. S3. Measurement of optical coherence time of Er^{3+}:$LiNbO_3$. (**A**) Experimental setup for measuring two-pulse photon echoes. CW laser: continuous-wave laser; AOM: acousto-optic modulator; VOA: variable optical attenuator; PBS: polarization beam splitter; PC: polarization controller; DR: dilution refrigerator; PD: photodetector; DOS: digital oscilloscope. The inset shows the pulses sequence. **(B)** Results of photon echo area as a function of the time delay t_{12} between the two pulses.

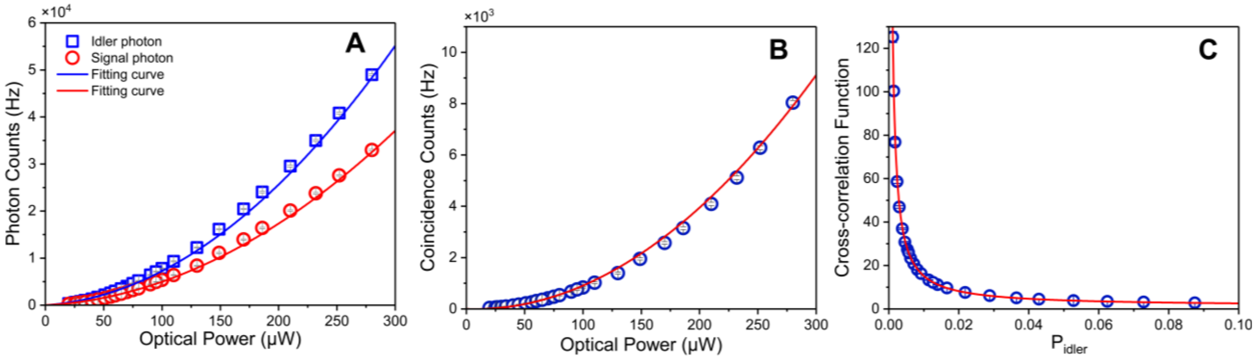

Fig. S4. Results of correlated photon pairs. (A) Single detection rates of signal photons and idler photons as a function of pump power. The red and blue solid lines are quadratic polynomial fitting curves. **(B)** Coincidences detection rate between signal photons and idler photons triggered by the system clock as a function of pump power. The red solid line is quadratic polynomial fitting curves. **(C)** Second-order cross-correlation function as a function of P_{idler}. The fitting curve is an inversely proportional function. All error bars are evaluated from the counts assuming Poissonian statistics.

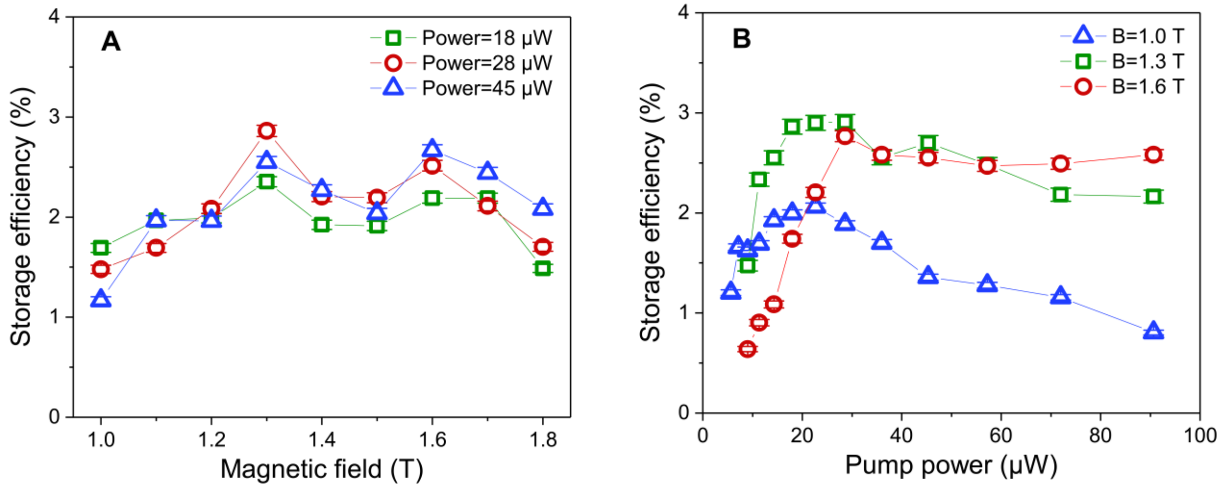

Fig. S5. Calibration measurements to estimate the internal storage efficiency with a storage time of 200 ns. (A) The internal storage efficiency as a function of the magnetic field. **(B)** The internal storage efficiency as a function of the pump power. Measurement with a pump time of 200 ms, a storage time of 200 ns and a storage bandwidth of 4 GHz.

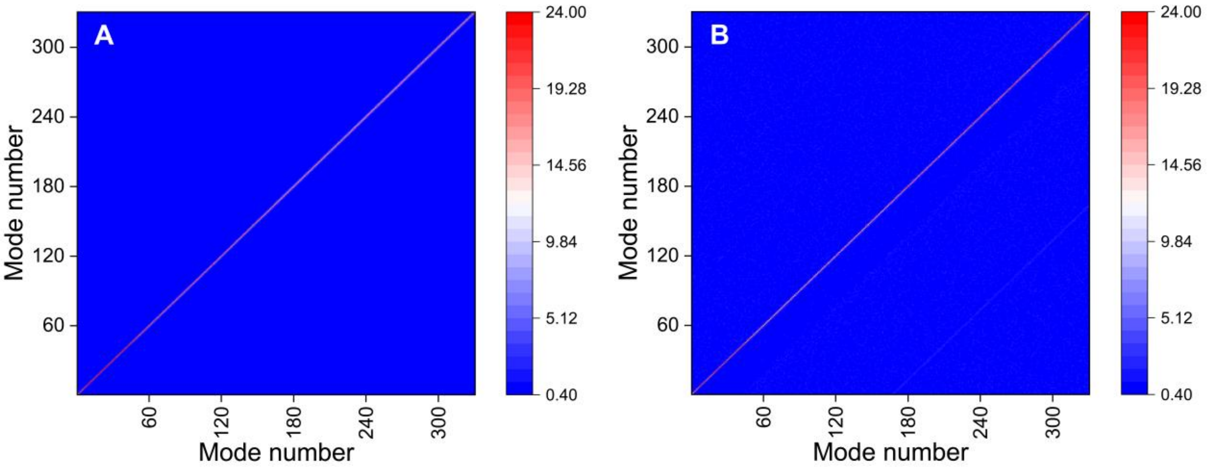

Fig. S6. 330×330 array of $g_{s,i}^{(2)}(t)$ among 330 temporal modes. (A) (B) The values of second-order cross-correlation function among 330 single photon modes before and after quantum storage, respectively.

Table S1.

Parameters of PPLN module.

Type of waveguide	RPE waveguide
Length of waveguide	50 mm
Poled period	19 μm
SHG normalized conversion efficiency	336.07%/W
Length of pigtail	20 cm
Input coupling efficiency of PPLN waveguide	55.51%
Output coupling efficiency of PPLN waveguide	77.89%

Table S2.

Measurement of internal storage efficiency (η) as a function of the storage time (Ts)

Ts (ns)	η (%)	Ts (ns)	η (%)
100	4.16±0.03	180	3.39±0.03
120	2.84±0.03	200	2.83±0.03
140	3.53±0.03	220	2.04±0.02
160	4.74±0.04	240	1.68±0.02

REFERENCES AND NOTES

1. A. I. Lvovsky, B. C. Sanders, W. Tittel, Optical quantum memory. *Nat. Photonics* **3**, 706–714 (2009).

2. C. Simon, M. Afzelius, J. Appel, A. Boyer de la Giroday, S. J. Dewhurst, N. Gisin, C. Y. Hu, F. Jelezko, S. Kröll, J. H. Müller, J. Nunn, E. S. Polzik, J. G. Rarity, H. De Riedmatten, W. Rosenfeld, A. J. Shields, N. Sköld, R. M. Stevenson, R. Thew, I. A. Walmsley, M. C. Weber, H. Weinfurter, J. Wrachtrup, R. J. Young, Quantum memories. *Eur. Phys. J. D* **58**, 1–22 (2010).

3. N. Sangouard, C. Simon, H. de Riedmatten, N. Gisin, Quantum repeaters based on atomic ensembles and linear optics. *Rev. Mod. Phys.* **83**, 33–80 (2011).

4. H. J. Kimble, The quantum internet. *Nature* **453**, 1023–1030 (2008).

5. S. Wehner, D. Elkouss, R. Hanson, Quantum internet: A vision for the road ahead. *Science* **362**, eaam9288 (2018).

6. C. Simon, Towards a global quantum network. *Nat. Photonics* **11**, 678–680 (2017).

7. S.-H. Wei, B. Jing, X.-Y. Zhang, J.-Y. Liao, C.-Z. Yuan, B.-Y. Fan, C. Lyu, D.-L. Zhou, Y. Wang, G.-W. Deng, H.-Z. Song, D. Oblak, G.-C. Guo, Q. Zhou, Towards real-world quantum networks: A review. *Laser Photonics Rev.* **16**, 2100219 (2022).

8. R. Uppu, F. T. Pedersen, Y. Wang, C. T. Olesen, C. Papon, X. Zhou, L. Midolo, S. Scholz, A. D. Wieck, A. Ludwig, P. Lodahl, Scalable integrated single-photon source. *Sci. Adv.* **6**, eabc8268 (2020).

9. N. C. Harris, D. Grassani, A. Simbula, M. Pant, M. Galli, T. Baehr-Jones, M. Hochberg, D. Englund, D. Bajoni, C. Galland, Integrated source of spectrally filtered correlated photons for large-scale quantum photonic systems. *Phys. Rev. X* **4**, 041047 (2014).

10. J. Wang, D. Bonneau, M. Villa, J. W. Silverstone, R. Santagati, S. Miki, T. Yamashita, M. Fujiwara, M. Sasaki, H. Terai, M. G. Tanner, C. M. Natarajan, R. H. Hadfield, J. L. O’Brien, M. G. Thompson, Chip-to-chip quantum photonic interconnect by path-polarization interconversion. *Optica* **3**, 407–413 (2016).

11. I. Pitsios, L. Banchi, A. S. Rab, M. Bentivegna, D. Caprara, A. Crespi, N. Spagnolo, S. Bose, P. Mataloni, R. Osellame, F. Sciarrino, Photonic simulation of entanglement growth and engineering after a spin chain quench. *Nat. Commun.* **8**, 1569 (2017).

12. B. Korzh, Q.-Y. Zhao, J. P. Allmaras, S. Frasca, T. M. Autry, E. A. Bersin, A. D. Beyer, R. M. Briggs, B. Bumble, M. Colangelo, G. M. Crouch, A. E. Dane, T. Gerrits, A. E. Lita, F. Marsili, G. Moody, C. Peña, E. Ramirez, J. D. Rezac, N. Sinclair, M. J. Stevens, A. E. Velasco, V. B. Verma, E. E. Wollman, S. Xie, D. Zhu, P. D. Hale, M. Spiropulu, K. L. Silverman, R. P. Mirin, S. W. Nam, A. G. Kozorezov, M. D. Shaw, K. K. Berggren, Demonstration of sub-3 ps temporal resolution with a superconducting nanowire single-photon detector. *Nat. Photonics* **14**, 250–255 (2020).

13. F. Marsili, V. B. Verma, J. A. Stern, S. Harrington, A. E. Lita, T. Gerrits, I. Vayshenker, B. Baek, M. D. Shaw, R. P. Mirin, S. W. Nam, Detecting single infrared photons with 93% system efficiency. *Nat. Photonics* **7**, 210–214 (2013).

14. L. You, X. Yang, Y. He, W. Zhang, D. Liu, W. Zhang, L. Zhang, L. Zhang, X. Liu, S. Chen, Z. Wang, X. Xie, Jitter analysis of a superconducting nanowire single photon detector. *Aip Adv.* **3**, 072135 (2013).

15. W. H. P. Pernice, C. Schuck, O. Minaeva, M. Li, G. N. Goltsman, A. V. Sergienko, H. X. Tang, High-speed and high-efficiency travelling wave single-photon detectors embedded in nanophotonic circuits. *Nat. Commun.* **3**, 1325 (2012).

16. L. You, Superconducting nanowire single-photon detectors for quantum information. *Nanophotonics* **9**, 2673–2692 (2020).

17. N. Sinclair, D. Oblak, C. W. Thiel, R. L. Cone, W. Tittel, Properties of a rare-earth-ion-doped waveguide at sub-Kelvin temperatures for quantum signal processing. *Phys. Rev. Lett.* **118**, 100504 (2017).

18. V. Dierolf, A. B. Kutsenko, A. Ostendorf, C. Sandmann, Spectral line broadening mechanism of Er^{3+} transitions in $Er:Ti:LiNbO_3$ channel waveguides. *Appl. Phys. B* **73**, 443–448 (2001).

19. N. Sinclair, E. Saglamyurek, M. George, R. Ricken, C. La Mela, W. Sohler, W. Tittel, Spectroscopic investigations of a Ti:Tm:$LiNbO_3$ waveguide for photon-echo quantum memory. *J. Lumin.* **130**, 1586–1593 (2010).

20. E. Miyazono, T. Zhong, I. Craiciu, J. M. Kindem, A. Faraon, Coupling of erbium dopants to yttrium orthosilicate photonic crystal cavities for on-chip optical quantum memories. *Appl. Phys. Lett.* **108**, 011111 (2016).

21. R. R. Gattass, E. Mazur, Femtosecond laser micromachining in transparent materials. *Nat. Photonics* **2**, 219–225 (2008).

22. E. Saglamyurek, N. Sinclair, J. Jin, J. A. Slater, D. Oblak, F. Bussières, M. George, R. Ricken, W. Sohler, W. Tittel, Broadband waveguide quantum memory for entangled photons. *Nature* **469**, 512–515 (2011).

23. E. Saglamyurek, N. Sinclair, J. Jin, J. A. Slater, D. Oblak, F. Bussières, M. George, R. Ricken, W. Sohler, W. Tittel, Conditional detection of pure quantum states of light after storage in a Tm-doped waveguide. *Phys. Rev. Lett.* **108**, 083602 (2012).

24. M. F. Askarani, M. L. G. Puigibert, T. Lutz, V. B. Verma, M. D. Shaw, S. W. Nam, N. Sinclair, D. Oblak, W. Tittel, Storage and reemission of heralded telecommunication-wavelength photons using a crystal waveguide. *Phys. Rev. Appl.* **11**, 054056 (2019).

25. D.-C. Liu, P.-Y. Li, T.-X. Zhu, L. Zheng, J.-Y. Huang, Z.-Q. Zhou, C.-F. Li, G.-C. Guo, On-demand storage of photonic qubits at telecom wavelengths. *Phys. Rev. Lett.* **129**, 210501 (2022).

26. I. Craiciu, M. Lei, J. Rochman, J. M. Kindem, J. G. Bartholomew, E. Miyazono, T. Zhong, N. Sinclair, A. Faraon, Nanophotonic quantum storage at telecommunication wavelength. *Phys. Rev. Appl.* **12**, 024062 (2019).

27. I. Craiciu, M. Lei, J. Rochman, J. G. Bartholomew, A. Faraon, Multifunctional on-chip storage at telecommunication wavelength for quantum networks. *Optica* **8**, 114–121 (2021).

28. G. Corrielli, A. Seri, M. Mazzera, R. Osellame, H. de Riedmatten, Integrated optical memory based on laser-written waveguides. *Phys. Rev. Appl.* **5**, 054013 (2016).

29. N. Sinclair, E. Saglamyurek, H. Mallahzadeh, J. A. Slater, M. George, R. Ricken, M. P. Hedges, D. Oblak, C. Simon, W. Sohler, W. Tittel, Spectral multiplexing for scalable quantum photonics using an atomic frequency comb quantum memory and feed-forward control. *Phys. Rev. Lett.* **113**, 053603 (2014).

30. A. Seri, D. Lago-Rivera, A. Lenhard, G. Corrielli, R. Osellame, M. Mazzera, H. de Riedmatten, Quantum storage of frequency-multiplexed heralded single photons. *Phys. Rev. Lett.* **123**, 080502 (2019).

31. M.-X. Su, T.-X. Zhu, C. Liu, Z.-Q. Zhou, C.-F. Li, G.-C. Guo, On-demand multimode optical storage in a laser-written on-chip waveguide. *Phys. Rev. A* **105**, 052432 (2022).

32. T. Zhong, J. M. Kindem, J. G. Bartholomew, J. Rochman, I. Craiciu, E. Miyazono, M. Bettinelli, E. Cavalli, V. Verma, S. W. Nam, F. Marsili, M. D. Shaw, A. D. Beyer, A. Faraon, Nanophotonic rare-earth quantum memory with optically controlled retrieval. *Science* **357**, 1392–1395 (2017).

33. T.-X. Zhu, C. Liu, L. Zheng, Z.-Q. Zhou, C.-F. Li, G.-C. Guo, Coherent optical memory based on a laser-written on-chip waveguide. *Phys. Rev. Appl.* **14**, 054071 (2020).

34. C. Liu, Z.-Q. Zhou, T.-X. Zhu, L. Zheng, M. Jin, X. Liu, P.-Y. Li, J.-Y. Huang, Y. Ma, T. Tu, T.-S. Yang, C.-F. Li, G.-C. Guo, Reliable coherent optical memory based on a laser-written waveguide. *Optica* **7**, 192–197 (2020).

35. C. Liu, T.-X. Zhu, M.-X. Su, Y.-Z. Ma, Z.-Q. Zhou, C.-F. Li, G.-C. Guo, On-demand quantum storage of photonic qubits in an on-chip waveguide. *Phys. Rev. Lett.* **125**, 260504 (2020).

36. A. Seri, G. Corrielli, D. Lago-Rivera, A. Lenhard, H. de Riedmatten, R. Osellame, M. Mazzera, Laser-written integrated platform for quantum storage of heralded single photons. *Optica* **5**, 934–941 (2018).

37. J. V. Rakonjac, G. Corrielli, D. Lago-Rivera, A. Seri, M. Mazzera, S. Grandi, R. Osellame, H. de Riedmatten, Storage and analysis of light-matter entanglement in a fiber-integrated system. *Sci. Adv.* **8**, eabn3919 (2022).

38. T.-X. Zhu, C. Liu, M. Jin, M.-X. Su, Y.-P. Liu, W.-J. Li, Y. Ye, Z.-Q. Zhou, C.-F. Li, G.-C. Guo, On-demand integrated quantum memory for polarization qubits. *Phys. Rev. Lett.* **128**, 180501 (2022).

39. S. Dutta, Y. Zhao, U. Saha, D. Farfurnik, E. A. Goldschmidt, E. Waks, An atomic frequency comb memory in rare-earth doped thin-film lithium niobate, in *Conference on Lasers and Electro-Optics FF3K.7* (Optica Publishing Group, 2022).

40. E. Saglamyurek, M. Grimau Puigibert, Q. Zhou, L. Giner, F. Marsili, V. B. Verma, S. Woo Nam, L. Oesterling, D. Nippa, D. Oblak, W. Tittel, A multiplexed light-matter interface for fibre-based quantum networks. *Nat. Commun.* **7**, 11202 (2016).

41. E. Saglamyurek, J. Jin, V. B. Verma, M. D. Shaw, F. Marsili, S. W. Nam, D. Oblak, W. Tittel, Quantum storage of entangled telecom-wavelength photons in an erbium-doped optical fibre. *Nat. Photonics* **9**, 83–87 (2015).

42. J. Jin, E. Saglamyurek, M. L. G. Puigibert, V. Verma, F. Marsili, S. W. Nam, D. Oblak, W. Tittel, Telecom-wavelength atomic quantum memory in optical fiber for heralded polarization qubits. *Phys. Rev. Lett.* **115**, 140501 (2015).

43. B. Zhang, L. Li, L. Wang, F. Chen, Second harmonic generation in femtosecond laser written lithium niobate waveguides based on birefringent phase matching. *Opt. Mater.* **107**, 110075 (2020).

44. C. W. Thiel, N. Sinclair, W. Tittel, R. L. Cone, Tm^{3+}:Y3Ga_5O_{12} Materials for spectrally multiplexed quantum memories. *Phys. Rev. Lett.* **113**, 160501 (2014).

45. M. F. Askarani, T. Lutz, M. G. Puigibert, N. Sinclair, D. Oblak, W. Tittel, Persistent atomic frequency comb based on Zeeman sub-levels of an erbium-doped crystal waveguide. *J. Opt. Soc. Am. B* **37**, 352–358 (2020).

46. C. W. Thiel, R. M. Macfarlane, T. Böttger, Y. Sun, R. L. Cone, W. R. Babbitt, Optical decoherence and persistent spectral hole burning in Er^{3+}:$LiNbO_3$. *J. Lumin.* **130**, 1603–1609 (2010).

47. Z. Zhang, C. Yuan, S. Shen, H. Yu, R. Zhang, H. Wang, H. Li, Y. Wang, G. Deng, Z. Wang, L. You, Z. Wang, H. Song, G. Guo, Q. Zhou, High-performance quantum entanglement generation via cascaded second-order nonlinear processes. *npj Quantum Inform* **7**, 123 (2021).

48. S.-H. Wei, B. Jing, X.-Y. Zhang, J.-Y. Liao, H. Li, L.-X. You, Z. Wang, Y. Wang, G.-W. Deng, H.-Z. Song, D. Oblak, G.-C. Guo, Q. Zhou, Storage of 1650 modes of single photons at telecom wavelength. arXiv:2209.00802 [quant-ph] (2022).

49. H. de Riedmatten, M. Afzelius, M. U. Staudt, C. Simon, N. Gisin, A solid-state light–Matter interface at the single-photon level. *Nature* **456**, 773–777 (2008).

50. S. Fasel, O. Alibart, S. Tanzilli, P. Baldi, A. Beveratos, N. Gisin, H. Zbinden, High-quality asynchronous heralded single-photon source at telecom wavelength. *New J. Phys.* **6**, 163 (2004).

51. P. R. Tapster, J. G. Rarity, Photon statistics of pulsed parametric light. *J. Mod. Optic.* **45**, 595–604 (1998).

52. Y. F. Pu, N. Jiang, W. Chang, H. X. Yang, C. Li, L. M. Duan, Experimental realization of a multiplexed quantum memory with 225 individually accessible memory cells. *Nat. Commun.* **8**, 15359 (2017).

53. D. G. Matei, T. Legero, S. Häfner, C. Grebing, R. Weyrich, W. Zhang, L. Sonderhouse, J. M. Robinson, J. Ye, F. Riehle, U. Sterr, 1.5 μm lasers with sub-10 mHz linewidth. *Phys. Rev. Lett.* **118**, 263202 (2017).

54. V. Snigirev, A. Riedhauser, G. Lihachev, M. Churaev, J. Riemensberger, R. N. Wang, A. Siddharth, G. Huang, C. Möhl, Y. Popoff, U. Drechsler, D. Caimi, S. Hönl, J. Liu, P. Seidler, T. J. Kippenberg, Ultrafast tunable lasers using lithium niobate integrated photonics. *Nature* **615**, 411–417 (2023).

55. J. Etesse, A. Holzäpfel, A. Ortu, M. Afzelius, Optical and spin manipulation of non-Kramers rare-earth ions in a weak magnetic field for quantum memory applications. *Phys. Rev. A* **103**, 022618 (2021).

56. Y.-Z. Ma, M. Jin, D.-L. Chen, Z.-Q. Zhou, C.-F. Li, G.-C. Guo, Elimination of noise in optically rephased photon echoes. *Nat. Commun.* **12**, 4378 (2021).

57. M. Afzelius, C. Simon, Impedance-matched cavity quantum memory. *Phys. Rev. A* **82**, 022310 (2010).

58. M. Sabooni, Q. Li, S. Kröll, L. Rippe, Efficient quantum memory using a weakly absorbing sample. *Phys. Rev. Lett.* **110**, 133604 (2013).

59. S. R. Hastings-Simon, M. U. Staudt, M. Afzelius, P. Baldi, D. Jaccard, W. Tittel, N. Gisin, Controlled Stark shifts in Er^{3+}-doped crystalline and amorphous waveguides for quantum state storage. *Opt. Commun.* **266**, 716–719 (2006).

60. M. Gündoğan, P. M. Ledingham, K. Kutluer, M. Mazzera, H. de Riedmatten, Solid state spin-wave quantum memory for time-bin qubits. *Phys. Rev. Lett.* **114**, 230501 (2015).

61. P. Jobez, C. Laplane, N. Timoney, N. Gisin, A. Ferrier, P. Goldner, M. Afzelius, Coherent spin control at the quantum level in an ensemble-based optical memory. *Phys. Rev. Lett.* **114**, 230502 (2015).

62. M. Rančić, M. P. Hedges, R. L. Ahlefeldt, M. J. Sellars, Coherence time of over a second in a telecom-compatible quantum memory storage material. *Nat. Phys.* **14**, 50–54 (2018).

63. M. Afzelius, C. Simon, H. de Riedmatten, N. Gisin, Multimode quantum memory based on atomic frequency combs. *Phys. Rev. A* **79**, 052329 (2009).

64. C. Simon, H. de Riedmatten, M. Afzelius, N. Sangouard, H. Zbinden, N. Gisin, Quantum repeaters with photon pair sources and multimode memories. *Phys. Rev. Lett.* **98**, 190503 (2007).

65. J. Dajczgewand, "Optical memory in an erbium doped crystal: Efficiency, bandwidth and noise studies for quantum memory applications," thesis, Université Paris Saclay (COmUE) (2015).

66. L. M. Johnson, C. H. Cox, Serrodyne optical frequency translation with high sideband suppression. *J. Lightwave Technol.* **6**, 109–112 (1988).

67. W. B. Mims, Phase memory in electron spin echoes, lattice relaxation effects in $CaWO_4$: Er, Ce, Mn. *Phys. Rev.* **168**, 370–389 (1968).

68. E. Saglamyurek, T. Lutz, L. Veissier, M. P. Hedges, C. W. Thiel, R. L. Cone, W. Tittel, Efficient and long-lived Zeeman-sublevel atomic population storage in an erbium-doped glass fiber. *Phys. Rev. B* **92**, 241111 (2015).

69. N. Sinclair, D. Oblak, E. Saglamyurek, R. L. Cone, C. W. Thiel, W. Tittel, Optical coherence and energy-level properties of a Tm^{3+}-doped $LiNbO_3$ waveguide at subkelvin temperatures. *Phys. Rev. B* **103**, 134105 (2021).
